# Fibrodysplasia Ossificans Progressiva: A Case Report From the UAE

**DOI:** 10.7759/cureus.105022

**Published:** 2026-03-11

**Authors:** Tabarak M Al Karam, Batool M Alkaram, Rafah Z Basil, Safwan A Alzaghal, Abdalla Ahmed

**Affiliations:** 1 General Practice, University of Sharjah, Sharjah, ARE; 2 Internal Medicine, University of Sharjah, Sharjah, ARE; 3 Internal Medicine, Al Qassimi Hospital, Sharjah, ARE

**Keywords:** cellulitis, fibrodysplasia ossificans progressiva (fop), hallux valgus, non-traumatic myositis ossificans, ultrarare disorders

## Abstract

Fibrodysplasia ossificans progressiva (FOP) is an ultra-rare autosomal dominant disorder characterized by congenital malformation of the great toes and progressive heterotopic ossification of soft tissues, caused by mutations in the ACVR1 gene. Flare-ups are painful inflammatory episodes that may occur spontaneously or following minor trauma. The estimated incidence is rare, and to our knowledge, a genetically unconfirmed case has previously been reported in the United Arab Emirates.

We report a 15-year-old Sudanese girl with genetically confirmed FOP, diagnosed at two years of age. She initially presented in infancy with painful shoulder restriction, followed by recurrent trauma- and infection-triggered flare-ups. Progressive heterotopic ossification led to jaw ankylosis, hip involvement with gait impairment, and eventual wheelchair dependence. During her most recent admission, she presented with painful right forearm swelling that was managed as cellulitis; this was later recognized as an FOP flare-up. Corticosteroid therapy was not initiated because she presented six days after symptom onset, whereas current recommendations support administration within 24 hours of flare-up onset. Management has primarily consisted of supportive care, physiotherapy for mobility preservation, and family education focused on trigger avoidance and early presentation to medical care at the onset of known flare-ups.

To our knowledge, this case represents the first genetically confirmed reported case of FOP in the region. It underscores the importance of early recognition of flare-ups and timely presentation to optimize management and improve patient education in this rare condition.

## Introduction

Fibrodysplasia ossificans progressiva (FOP) is a rare autosomal dominant connective tissue disorder characterized by progressive heterotopic ossification of extraskeletal tissue and congenital malformation of the great toes. First clinically described in the 17th century and genetically characterized in 2006 [1,2], FOP is now definitively diagnosed through genetic identification of pathogenic ACVR1 mutations [3]. Disease progression may arise spontaneously or be precipitated by minor trauma, leading to functional impairment. Management is largely preventative and supportive, focusing on trauma avoidance and flare control [3]. With an incidence of 1 in every 2,000,000 individuals [4], to our knowledge, only one other case report has been published in the United Arab Emirates [5].

In this report, we present a 15-year-old girl with painful forearm swelling, highlighting the diagnostic challenge of distinguishing inflammatory flare-ups from soft tissue infection in a genetically confirmed case within a low-prevalence region and adding to the limited literature on FOP in the region.

## Case presentation

A 15-year-old Sudanese female presented to the Emergency Department of Al Qassimi Hospital, Sharjah, UAE, on August 15, 2025, with acute, progressively worsening pain of the right forearm and hand, accompanied by swelling of six days’ duration. The pain was sudden in onset, sharp in character, and progressively extended from the forearm to involve the wrist and hand. Swelling developed shortly after pain onset and followed a similar distribution. The episode was associated with stiffness and marked restriction of the range of motion at the elbow and wrist. No clear precipitating trauma was identified, although repetitive mechanical strain related to school activities was considered a possible trigger. Medical attention was sought as the swelling became increasingly pronounced and functionally limiting.

**Figure 1:**
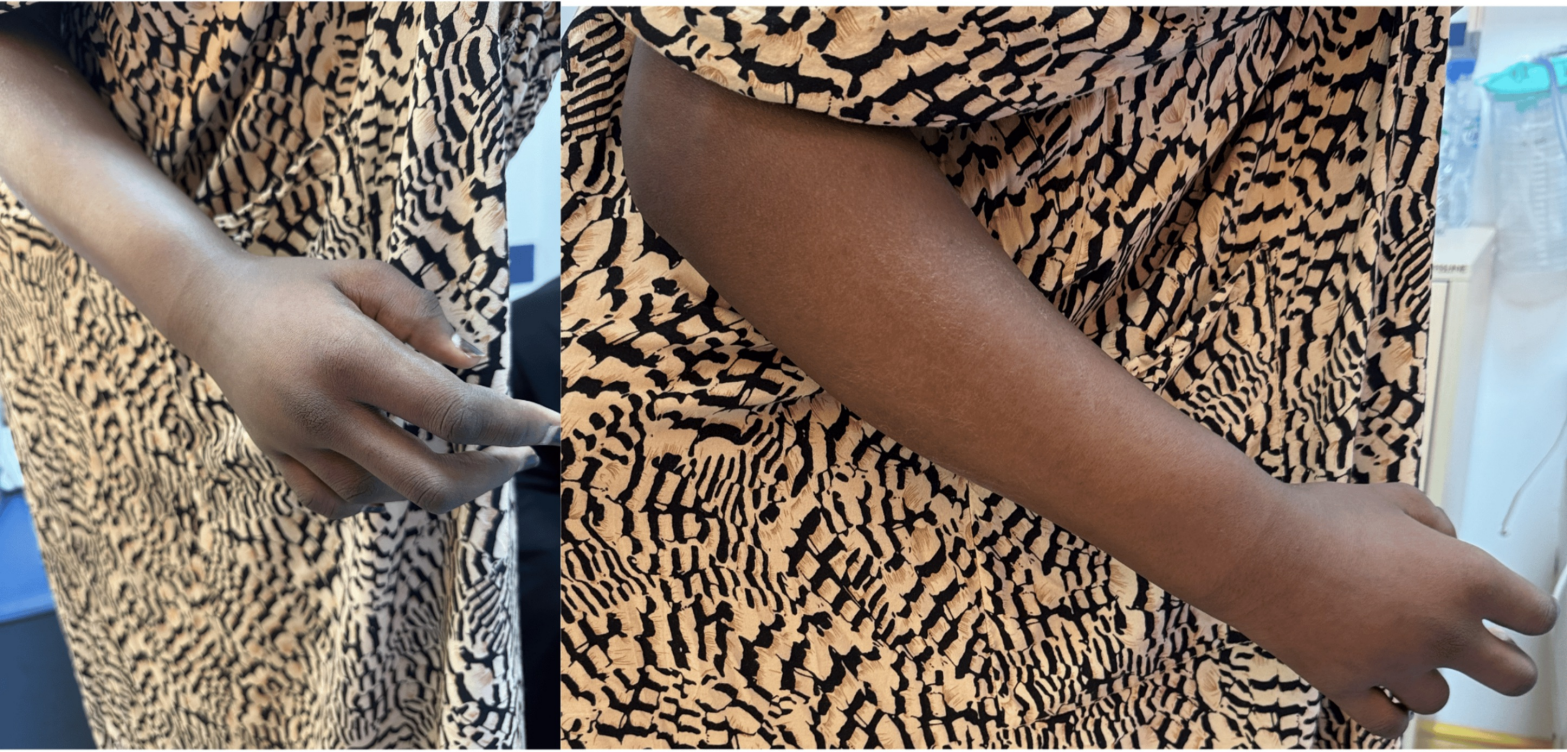
Photographs of the hand and arm

On examination, shortening of the fourth and fifth metacarpals was observed; the right upper limb was fixed in a mid-prone position with approximately 90° elbow flexion. The forearm was markedly swollen with tense, shiny overlying skin, without erythema, wounds, or visible skin breach. Range of motion was severely restricted at the elbow, wrist, and metacarpophalangeal joints, while the interphalangeal joints were relatively spared (Figure 1).

**Figure 2:**
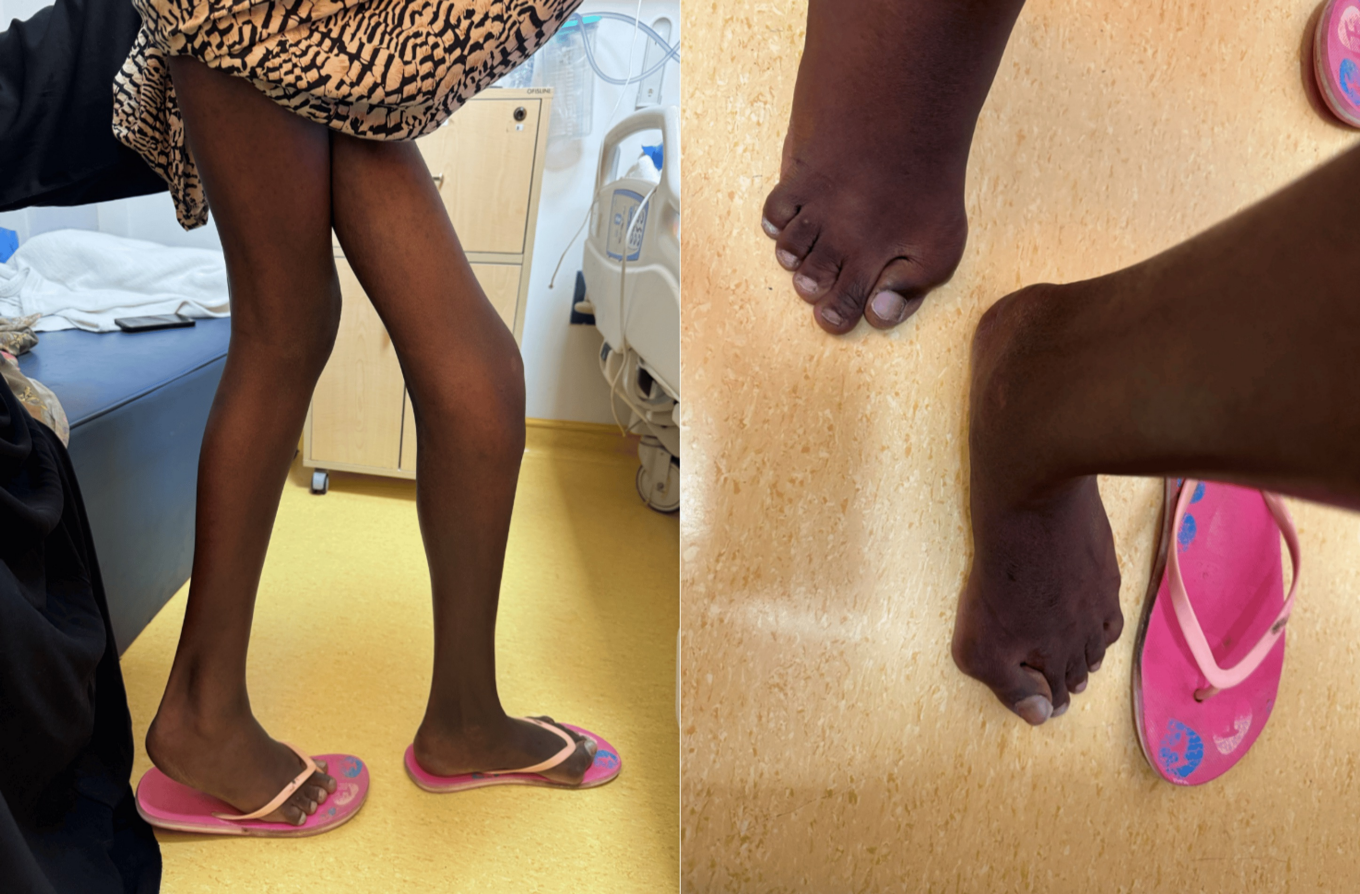
Photographs of lateral knee joints and superior metatarsophalangeal joints First metatarsophalangeal joints showing hallux valgus.

Both shoulders demonstrated fixed deformity with marked limitation of movement, more pronounced on the right, with prominent scapular protrusion consistent with periscapular heterotopic ossification. The left upper limb retained partial distal motor function despite severe proximal restriction. Temporomandibular joint involvement was evident, with limited mouth opening contributing to feeding difficulty. The lower limbs revealed fixed hip and knee deformities with right-sided pelvic tilt and an abnormal right-leaning gait, reflecting asymmetric hip involvement. Hip abduction was restricted on both active and passive examination, suggesting a structural rather than pain-causing limitation. Lumbar spine mobility was reduced with compensatory hyperlordosis. The right knee was medially fixed, resulting in leg crossing at rest. Bilateral congenital hallux valgus with associated macrodactyly was noted (Figure 2), supporting the diagnosis of classic FOP. The patient primarily mobilized using a wheelchair to avoid trauma and prevent the risk of flare-up progression.

Initial symptoms were first noted at one year, when the patient developed pain with shoulder elevation during handling, initially misdiagnosed as an axillary soft tissue infection. At two years, she developed progressive bilateral limitation of shoulder abduction. Radiographic evaluation demonstrated ectopic calcifications, and subsequent genetic testing confirmed the diagnosis of FOP. Both shoulders were involved: partial functional improvement occurred on the left, while the right remained limited.

Over subsequent years, the patient experienced recurrent flare-ups characterized by painful inflammatory soft tissue swellings that progressed into irreversible ossification and cumulative functional impairment. Minor trauma was identified as the most consistent precipitating factor, while infections were less frequently associated.

Family history revealed nonconsanguineous parents with no known history of FOP or other hereditary skeletal disorders. A paternal aunt was noted to have hallux valgus; however, no family members exhibited features suggestive of heterotopic ossification or musculoskeletal deformity.

During the current admission, the patient was afebrile and hemodynamically stable, and no leukocytosis was observed. Inflammatory markers were minimally elevated (CRP 1.4 mg/L), and autoimmune screening, including ANA, RNP/Sm, and Scl-70 antibodies, was negative.

**Figure 3:**
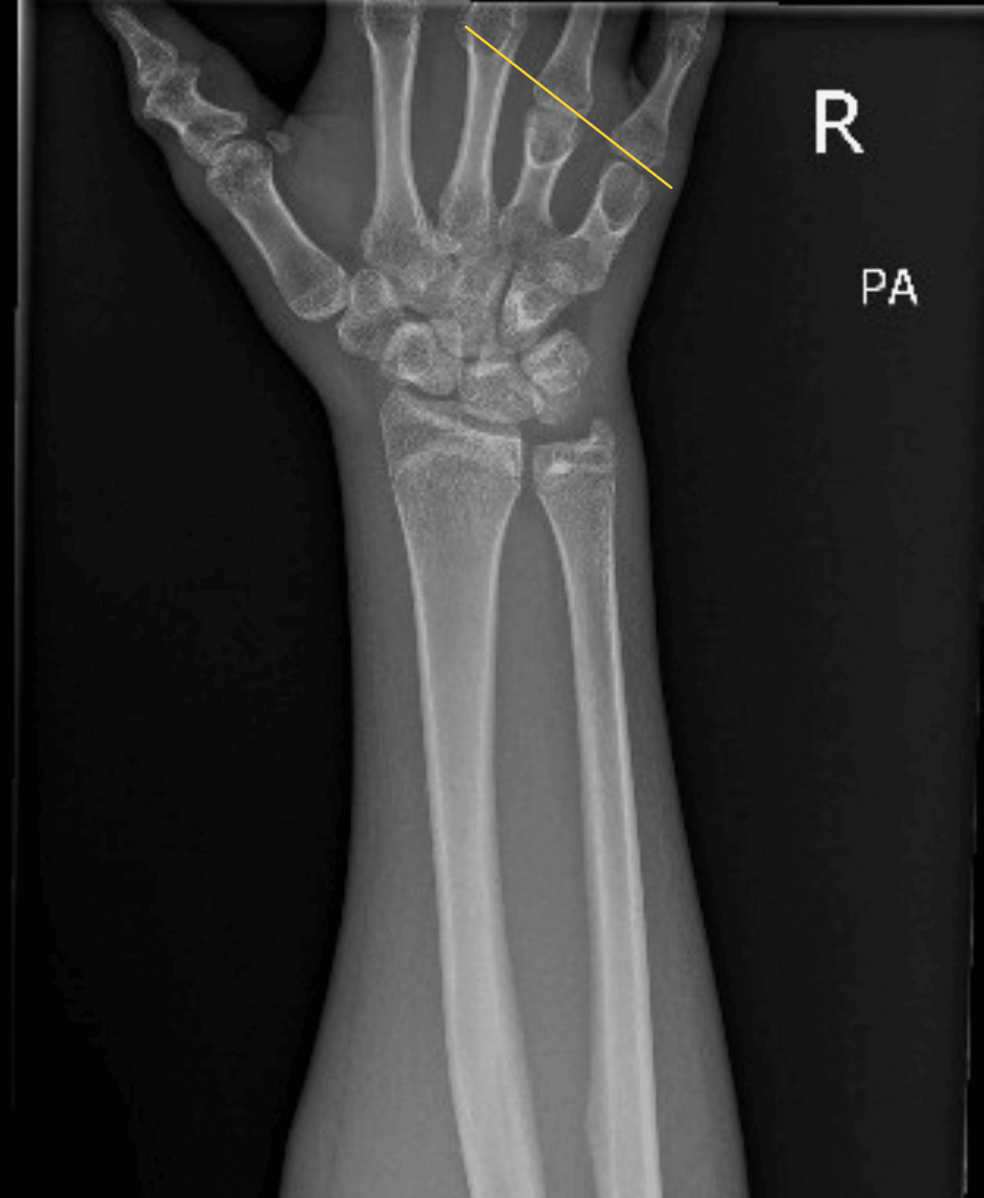
Posteroanterior radiograph of the right wrist Line following the head of the fourth and fifth metacarpals and intersecting the third, showing the metacarpal sign.

Posteroanterior radiograph of the right wrist demonstrating preserved alignment of the distal radius and ulna without evidence of acute fracture or aggressive osseous pathology. Congenital shortening of the fourth and fifth metacarpals is present. No radiographic signs of osteomyelitis, periosteal reaction, or soft tissue gas are identified. No soft tissue gas or calcified mass is visualized at this stage, consistent with an early inflammatory flare rather than mature heterotopic ossification or infection (Figure 3). 

**Figure 4:**
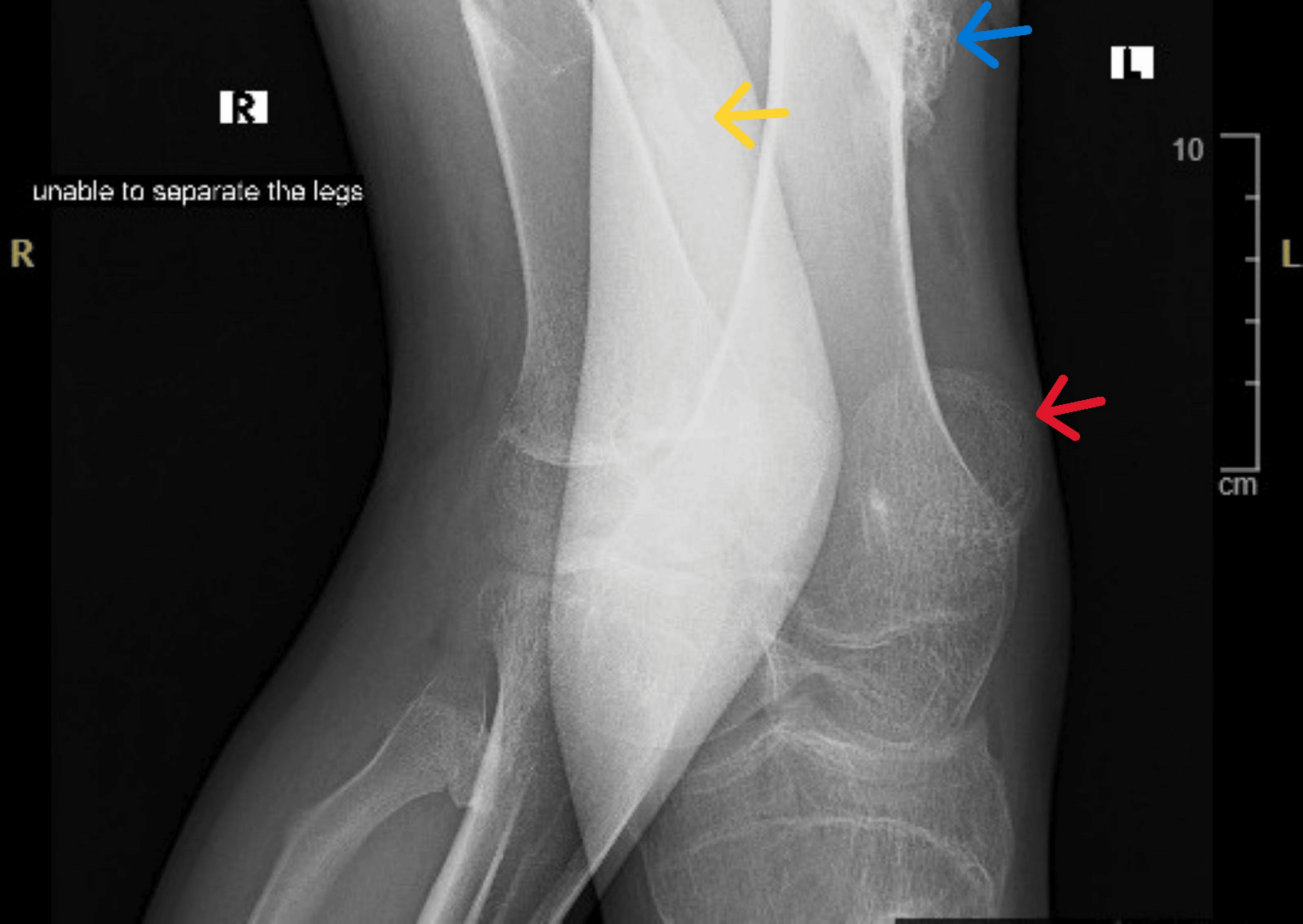
Radiograph of the knee joints Blue arrow shows ossified masses with cortical margins and internal trabeculation; yellow arrow shows sheet-like heterotopic ossification; red arrow shows an osteochondroma-like lesion.

Bilateral lower limb radiographs show extensive, mature, sheet-like heterotopic ossification within the soft tissues of both thighs, following a longitudinal muscle-plane distribution. The ossified masses show well-defined cortical margins and internal trabeculation, consistent with mature extraskeletal bone formation rather than acute inflammatory change. A well-circumscribed bony outgrowth arising from the distal shaft of the left femur, compatible with an osteochondroma-like lesion, is also observed. The patient was unable to separate the lower limbs during imaging, reflecting functional ankylosis secondary to mature ectopic bone formation (Figure 4).

**Figure 5:**
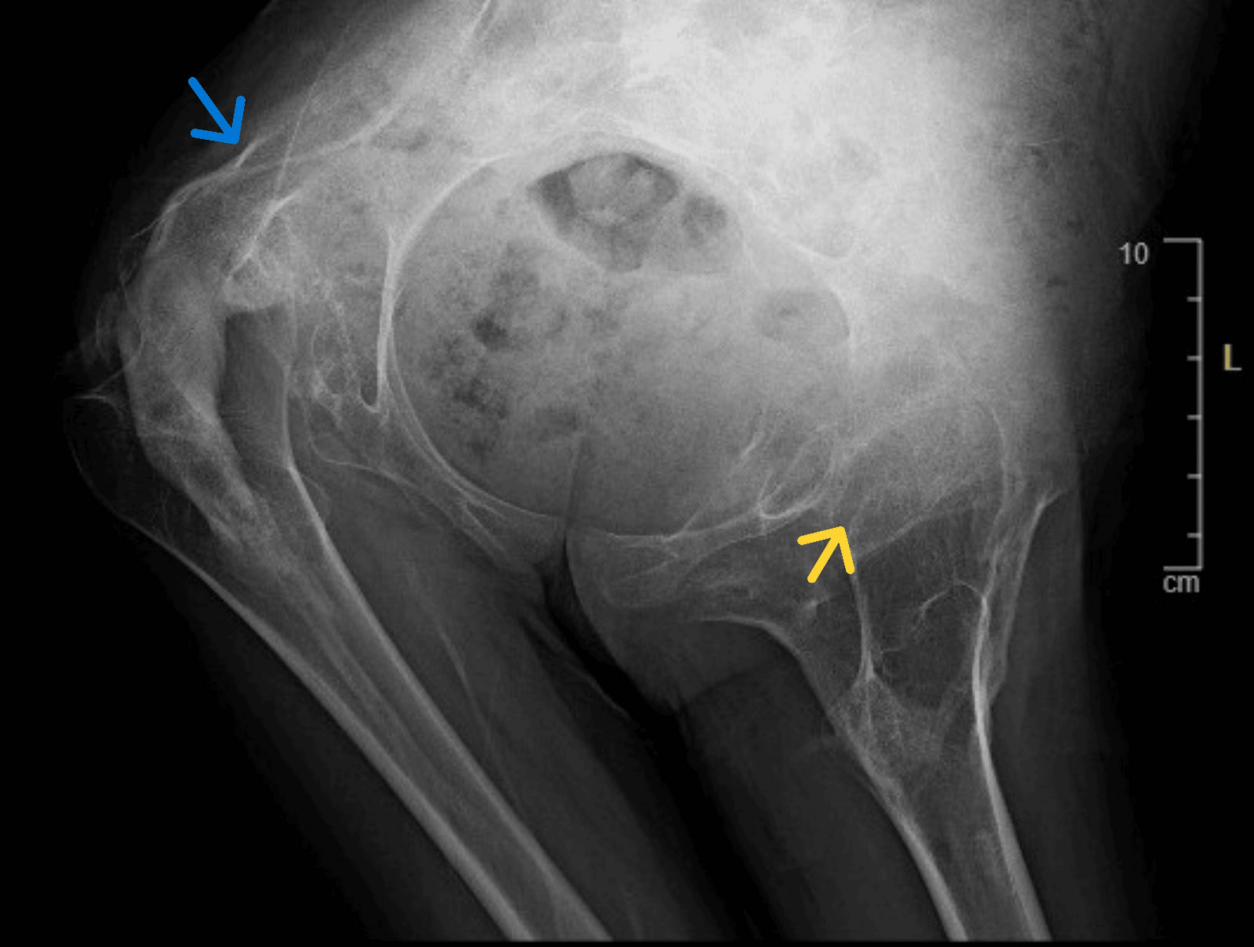
Pelvic radiograph Blue arrow points at periarticular ossification surrounding the hip joint, and yellow arrow shows the relatively preserved femoroacetabular joint space.

Pelvic radiograph demonstrating extensive, mature heterotopic ossification surrounding both hip joints, with well-formed extraskeletal bone bridging periarticular soft tissues. The femoroacetabular joint spaces remain relatively preserved, indicating mechanical restriction of hip motion due to heterotopic ossification rather than primary intra-articular pathology (Figure 5).

**Figure 6:**
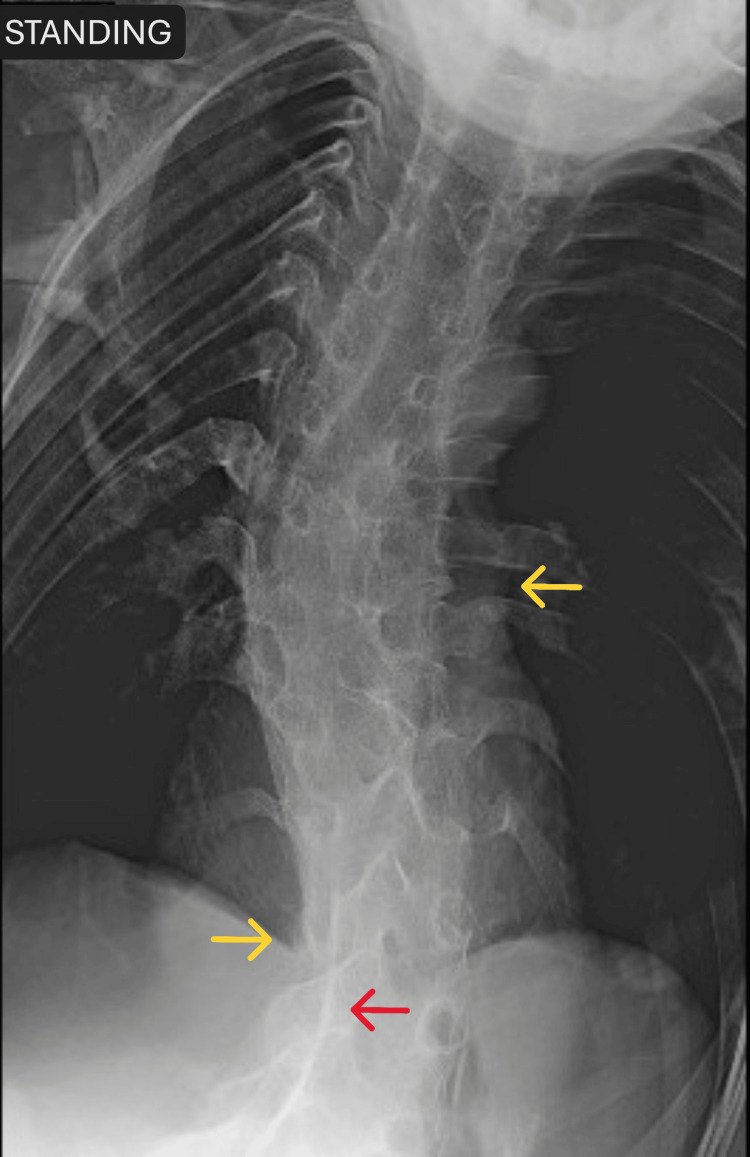
Spine radiograph Red arrow shows bridging heterotopic ossification, and yellow arrows show dorsolumbar kyphoscoliosis.

Similarly, spinal radiographs demonstrated extensive, mature heterotopic ossification involving the paraspinal soft tissues, with bridging extraskeletal bone across multiple posterior spinal elements, resulting in marked reduction of segmental spinal mobility. The ossification is extra-articular and does not originate from the vertebral endplates or sacroiliac joints, distinguishing it from inflammatory spondyloarthropathies. An associated dorsolumbar kyphoscoliosis is present, with right thoracic and left lumbar convexity, likely reflecting chronic asymmetric restriction of spinal motion due to heterotopic bone formation (Figure 6).

Given an initial working diagnosis of cellulitis, the patient was treated with oral amoxicillin/clavulanic acid (875 mg/125 mg) for 10 days, along with sodium aescinate and tramadol 50 mg for analgesia. After revision of the case, FOP flare-up was more likely, but systemic corticosteroids were not initiated due to delayed hospital presentation (beyond 24 hours of the onset of symptoms); patient education was provided. She had previously undergone physiotherapy following traumatic episodes, with partial but transient functional improvement. No disease-modifying therapy had been initiated, and palovarotene was not available at the treating institution. Referral for specialized care and treatment abroad had been considered but was interrupted due to socioeconomic and public health-related travel constraints.

## Discussion

FOP is an ultra-rare genetic disorder, with an estimated prevalence of 0.5-0.88 per million [6], likely underrecognized due to limited access to molecular diagnostics and rare disease registries [7]. Only one prior case has been reported in the United Arab Emirates, without genetic confirmation [5]. To our knowledge, the present case represents the first genetically confirmed FOP case in the United Arab Emirates and the second in the region.

Classic FOP results from a recurrent heterozygous mutation in the ACVR1 gene, most commonly c.617G>A (p.Arg206His) [6,8,9], as identified in our patient.

Clinically, the diagnosis is supported by congenital hallux valgus and progressive heterotopic ossification triggered by minor trauma [3]. Alternative diagnoses, such as progressive osseous heteroplasia, myositis ossificans traumatica, and Albright hereditary osteodystrophy, were excluded based on the presence of congenital toe malformations, episodic progression, and molecular confirmation. Biopsy was appropriately avoided. According to the FOP Registry, 53.5% of patients were initially misdiagnosed [7]. This carries significant risk, particularly when invasive diagnostic procedures could precipitate further ossification [7].

Differential diagnosis of acute forearm swelling

The absence of fever, minimal CRP elevation (1.4 mg/L), lack of skin breach, and a history of similar inflammatory episodes favored an FOP flare-up. Cellulitis typically presents with systemic features and marked inflammatory response, whereas FOP flare-ups manifest as deep, painful soft tissue swellings that may mimic infection in early stages. Although our patient was initially treated for presumed cellulitis, no invasive interventions were performed, and thus, no iatrogenic harm occurred. The case nonetheless illustrates how even established FOP patients may experience diagnostic ambiguity during acute presentations.

Management considerations

Standard flare management includes early administration of corticosteroids (prednisone 2 mg/kg/day for four days) within 24 hours of symptom onset, followed by symptomatic control with non-steroidal anti-inflammatory drugs (NSAIDs) and strict avoidance of further soft tissue trauma [10]. In this instance, corticosteroids were not administered because the patient presented beyond the recommended therapeutic window.

Palovarotene, the only approved disease-modifying therapy for FOP [10], was not initiated. The treating institution did not have access to the medication, and no publicly available data confirm its routine availability locally. The patient is not currently followed in a specialized FOP center, reflecting limitations in regional rare disease infrastructure and potentially restricting access to advanced therapies. Rapamycin remains investigational [11].

Comprehensive management requires multidisciplinary follow-up and preventive strategies, including avoidance of intramuscular injections, fall prevention, pulmonary monitoring, and adaptive rehabilitation [10]. The absence of specialized care in this case underscores ongoing structural gaps in rare disease management within low-prevalence settings.

## Conclusions

To our knowledge, this report describes the first genetically confirmed case of FOP in the United Arab Emirates. It emphasizes the diagnostic challenge of distinguishing flare-ups from soft tissue infection and highlights the implications of limited access to specialized care and disease-modifying therapies. Early clinical recognition, prompt genetic confirmation, structured referral pathways, and comprehensive patient education, on early flare-up identification, to ensure presentation within the therapeutic window, are essential to optimize management and prevent avoidable disease progression in rare genetic disorders such as FOP.
